# Megacities and the Environment

**DOI:** 10.1100/tsw.2002.103

**Published:** 2002-02-09

**Authors:** Ethan H. Decker, Scott Elliott, Felisa A. Smith

**Affiliations:** Department of Biology, University of New Mexico, Albuquerque, NM 87131, USA; Atmospheric and Climate Sciences Group, Los Alamos National Laboratory, Los Alamos, NM 87545, USA

**Keywords:** megacities, megacity biogeochemistry, urban succession, urban metabolism, atmospheric processes, earth system modeling, urban planning, metropolitan agglomerations, material consumption

## Abstract

The world’s 25 largest cities comprise only 4% of the global population, but they have substantial impacts on the environment at multiple scales. Here we review what is known of the biogeochemistry of these megacities. Climatic, demographic, and economic data show no patterns across cities, save that wealthier cities have lower growth rates. The flows of water, fuels, construction materials, and food are examined where data are available. Water, which by mass dwarfs the other inputs, is not retained in urban systems, whereas construction materials and food predominate in the urban infrastructure and the waste stream. Fuels are transformed into chemical wastes that have the most far-reaching and global impacts. The effects of megacity resource consumption on geologic, hydrologic, atmospheric, and ecological processes are explored at local, regional, and global scales. We put forth the concepts of urban metabolism and urban succession as organizing concepts for data collection, analysis, and synthesis on urban systems. We conclude that megacities are not the final stage of urban evolution; rather, the climax of urban development will occur at a global scale when human society is at steady state with resource supply rates.

## INTRODUCTION

Humans are the most dominant species on Earth. We consume an enormous amount of terrestrial and oceanic biotic productivity, we have transformed close to half of the earth's land surface, and we have severely altered global biogeochemical cycles[1,2,3]. Over the past century, our global population has quadrupled from 1.6 billion to 6 billion[4], while our total energy use has increased 16-fold to an estimated 355 exajoules (EJ) per year (1 EJ = $10^{18}$ J)[5]. Our use of materials, energy, and space is a heavy burden on the environment at local, regional, and global scales[6,7,8].

Urbanization has increased lockstep with energy and material use. Exactly what comprises an urban area depends on what definition is used, and there are currently no international standards for data collection on urban areas, cities, or metropolitan agglomerations[9]. Despite this lack of standardization, general global trends are apparent. Whereas 30% of the world's population lived in cities in 1950, nearly half of all people are urbanites today, and the proportion is expected to reach 60% by 2030[10]. The populations of Europe and North America are both over 75% urban, and some nations (e.g., Lebanon, the U.K., the Netherlands, Argentina) are nearly 90% urban. Furthermore, virtually all the expected growth in the global population will most likely occur in urban or urbanizing areas of the developing world.

Most urbanites live in cities of less than 100,000 inhabitants[10]. Currently there are over 330 cities with more than 1 million inhabitants and 23 urban agglomerations of 10 million people or more[11]. Comprising only 4.3% of the world population, these 23 megacities — whose functional populations extend beyond their official city limits — have a disproportionate impact on regional and global economies and environments (Table 1). Although the growth of these megacities is slowing, other cities will soon gain megacity status through a combination of outright growth of single cities and regional agglomeration of multiple large cities. Megacities offer the most striking examples of environmental, technical, social, and policy challenges that accompany intense urbanization. For this reason, it is revealing to study the structure, dynamics, and impacts of the world's megacities.

Many important questions can be addressed by studying megacities. How large can cities grow? Is city growth constrained by social processes or natural resource limitations, and at what scales? Are there basic energetic constraints or rules for the development of large metropolitan regions? What are the links and feedbacks between urban processes and global environmental process such as biogeochemical cycles? How is extensive urbanization affecting the flow of materials and energy around the world?

Herein we provide a brief review of megacities and the environment. We begin by describing common features of megacity structure and function. We discuss impacts and feedbacks between urban agglomerations and their environs at local, regional, and global scales. We develop the concepts of urban metabolism and urban succession as conceptual frameworks for understanding and investigating urban systems. We outline future directions in research and policy, and then we conclude with some comments about the notion of sustainability. An earlier review[12] provides a more extensive examination of material and energy flows through the 25 largest cities of the world.

## SOCIOECONOMICS AND BIOGEOCHEMISTRY

### Megacities Are Everywhere

The world's largest cities are mainly found in mid-latitude regions near large sources of water. With the exceptions of Moscow, Delhi, Mexico City, and Tehran, they are port towns close to large river mouths. However, beyond these generalizations, megacities have little in common regarding where they are situated. Topography, climate, and annual precipitation all vary considerably. For example, mean annual precipitation ranges from 25 mm in Cairo to 2,129 mm in Bombay, and monthly mean minimum temperature ranges from 26°C in Jakarta to -10°C in Moscow[12].

Demographic and economic features of the world's largest metropolitan areas are also highly variable. Megacities exist in small island nations (e.g., Tokyo and Manila), large, sparsely populated countries (e.g., Cairo and Buenos Aires), and global giants (e.g., Calcutta, New York,

**TABLE 1**
**Demographic and Socioeconomic Indicators of the World's Megacities[a]**

| City | 2000 Pop.(b) ($10^6$) | Growth Rate(b) ($year^{-1}$) | Area(c) ($km^2$) | Climate(g) | National GDP(h) (US$) | Solid waste generation(i) (tons/day) |
|---|---|---|---|---|---|---|
| Tokyo | 27.9 | 0.8 | 2,162 | temperate | 33,265 | 66,000 |
| Bombay | 18.1 | 3.7 | 603 | tropical | 402 | 5,000 |
| São Paulo | 17.8 | 1.6 | 8,000 | tropical | 4,930 | 20,200 |
| Shanghai | 17.2 | 2.6 | 6,300 | temperate | 745 | 12,000 |
| New York | 16.6 | 0.4 | 3,585 | cold | 28,789 | 13,000 |
| Mexico City | 16.4 | 0.9 | 2,500 | temperate | 4,265 | 15,046 |
| Beijing | 14.2 | 2.8 | 16,800 | cold | 745 | — |
| Jakarta | 14.1 | 4.1 | 5,500(d) | tropical | 1,055 | 13,400 |
| Lagos | 13.5 | 5.4 | — | tropical | 1,376 | 3,100 |
| Los Angeles | 13.1 | 1.2 | 16,600 | temperate | 28,789 | 20,360 |
| Calcutta | 12.7 | 1.6 | 1,295 | tropical | 402 | — |
| Tianjin | 12.4 | 2.9 | 11,305(e) | arid | 745 | 16,800 |
| Seoul | 12.3 | 1.1 | 1,650 | cold | 9,677 | — |
| Karachi | 12.1 | 4.1 | 3,530 | arid | 466 | — |
| Delhi | 11.7 | 3.3 | 591 | temperate | 402 | 12,000 |
| Buenos Aires | 11.4 | 0.7 | 7,000 | temperate | 9,070 | — |
| Manila | 10.8 | 3 | 636 | tropical | 1,151 | 6,500 |
| Cairo | 10.7 | 2.1 | 214 | arid | 1,168 | 4,800 |
| Osaka | 10.6 | 0 | — | temperate | 33,265 | — |
| Dhaka | 10.2 | 5.3 | — | tropical | 286 | 780 |
| Rio de Janeiro | 10.2 | 0.7 | 6,500 | tropical | 4,930 | 10,900 |
| Moscow | 9.3 | 0.1 | 994 | cold | 3,028 | 4,490 |
| Bangkok | 7.3 | 2.2 | 1,565 | tropical | 2,576 | 6,000 |
| London | 7.3 | 0 | 1,579 | temperate | 21,921 | — |
| Tehran | 7.3 | 1.5 | >600(f) | arid | 2,466 | — |
| Mean | 13.0 | 2.1 | 4,321 | | 7,837 | 13,550 |

(a) All values contain considerable uncertainty; (b) 2000 population is estimated[9]; (c) Area is UN Metropolitan Area[11]; (d) [129]; (e) [130]; (f) [131]; (g) [132]; (h) GDP, per capita 1997[13]; (i) [8].

Beijing). Whereas Buenos Aires constitutes 32% of Argentina's population of 36 million, Tianjin's 12.4 million residents comprise only 1% of China's population[4]. Population densities in these urban agglomerations are as low as 747/km$^2$ in Los Angeles (where there are two cars for every three residents) and as high as 45,000/km$^2$ in Cairo (where there is barely one car for every ten residents)[11]. National gross domestic product (GDP) for the 18 countries where megacities exist ranges from US$33,265 per capita in Japan to US$286 in Bangladesh, with a mean value of US$7,035 (Table 1)[13]. These nations run the gamut from highly developed to barely developed, varying in economic performance and global impact[14]. One regularity among megacities is that population growth rate decreases with increasing wealth; to use the above examples, Osaka and Dhaka are growing at 0 and 5.3%, respectively[13]. Beyond their massive population size, the major commonality among the world's largest cities is that they are voracious consumers of materials and energy.

## Megacities Are Voracious

Full material and energy budgets for megacities do not exist, but data can be found on a number of different urban material flows. It is clear that water dwarfs other inputs, comprising up to 90% of all mass entering cities. Megacities consume on average 1 megaton (Mt) of water daily, nearly all of which leaves the cities as treated or untreated wastewater[12]. Tokyo and Osaka consume 6.96 and 2.43 Mt/day, respectively[15], while Dhaka and Lagos consume 0.9 and 0.72 Mt, respectively[8]. By comparison, Mexico City consumes 10.124 Mt of fuel per *year*[11,16]. Wealthy cities such as Los Angeles have centralized water treatment and distribution facilities that serve virtually 100% of their population with clean water and sewerage (piped sewers). However, even these cities are facing water shortages. Tokyo has the most progressive approach for reducing water shortages, using a variety of on-site and municipal waste water reclamation programs that harvest rainwater and reuse urban runoff for nonpotable water needs[17,18,19]. While data are scarce for many developing megacities, piped water service is estimated to be as low as 15% (in Jakarta) and sewerage service as low as 2% (in Lagos)[8,20], with many megacities in the 40 to 60% range[21,22,23]. Municipal water systems are generally leaky, with loss rates such as 33% in Bombay, 37% in Seoul, and 49% in Bangkok[24]. In developing cities, water service is usually scarce in the urban periphery, where millions of residents in squatter settlements must get fresh water from trucks, neighborhood spigots, or rooftop harvesting[8,25].

Beyond water, material inputs to metropolitan regions include fuels, construction materials, and food. Because of the paucity of data at the city level, rates must often be estimated from nation-level statistics. For example, the U.S. material budget is 44.5% construction materials (of which 75% are aggregates), 38.2% fuels, 12.3% agricultural products, and 5.1% forestry products[26,27]. Urban areas will likely use more fuels, in particular petroleum products, and fewer agricultural products, as many are fed to livestock.

Roughly half of urban fuels are used for transportation. In Mexico City, which is perceived as the archetypal settlement of the next century, transportation accounts for 50% of all fossil fuel consumption[11,16]. In Bangkok, transport sector fuel use rose from 43 to 56% between 1973 and 1986[28]. Increasing fuel use in the transport sector parallels increases in auto use; since 1970, motor vehicles in Shanghai increased 13-fold[29]. Other urban fuel consumption includes industrial use and electricity generation. Megacities vary greatly as to the predominant fuel types in these sectors: oil in Jakarta and Manila; natural gas in Bangkok, Bombay, London, Mexico City, and Moscow; coal in Beijing, Shanghai, Calcutta, and Seoul; and hydroelectric in São Paulo and Los Angeles[11]. Importantly, many estimates on urban fuel use exclude biofuels such as wood, charcoal, and dung, which are commonly used by lower income groups in developing nations[12].

Much less information is available on food and construction material flows through megacities. Estimates for food inputs is difficult because the production and delivery system is extremely diffuse. One study of New York City found that 20,000 tons of food were consumed daily[30]. Half of this is eaten; the rest is solid waste, often rotting before it gets sold. Data for Shanghai provide one example of construction materials consumption. From 1990–1993, 20 million $m^2$ of floor space were built[31]. With an average mass of 450 $kg/m^2$[32], the new buildings added roughly 9.3 million tons of material to the city.

Unlike water and fuel, whose use transforms them to waste products that ultimately leave the city, the bulk mass of construction inputs and food remain in the city, either as useful infrastructure or as landfill. Solid waste generation averages 13,000 metric tons per day (t/d) in megacities, ranging from 780 t/d in Dhaka to 66,000 t/d in Tokyo (Table 1)[8,33]. In Mexico City, 43% of solid waste is food refuse, while the remainder is largely paper products, glass, and diapers[25]. However, in less developed megacities, less than half the population is served by municipal solid waste services[12]. Nearly 30% of Mexico City's waste ends up in unlined, unmanaged illegal dumps[25]. Waste management and recycling increase with increasing development. Remarkably, over 60% of Tokyo's waste stream is recycled[34]. Even so, the city has run out of space for landfills, and islands of waste are being built in Tokyo Bay[33].

Overall, megacities are voracious, growing physically and generating large quantities of waste products. They are the most poignant examples of modern human consumption, which has quadrupled since 1800 and increased 16-fold since the Neolithic era[35,36]. This vast influx of water, materials, fuel, and food results in a concomitant export of products and waste to adjoining and distant ecosystems. It is this combination of resource consumption and waste export by such large human systems that generates important environmental and social feedbacks between megacities and other places at local, regional, and global scales. The extent of a city's impact has been aptly called its "ecological footprint"[7,37]. It is only recently becoming clear how very large a megacity footprint is.

## ECOLOGICAL FOOTPRINTS

### Local Impacts

Metropolitan areas with over 10 million people (sometimes called "megalopolises") modify their own climate and suffer from deteriorated water and air quality. The largest cities become warm, impermeable, physically sinking environments. The combination of an increase in heat-absorbing surfaces (such as asphalt), increased surface area (from buildings), and increased heat production (due to fuel burning for industry, transportation, and heating of water and space) results in an increase in mean surface and air temperatures of several degrees Celsius[38,39,40,41]. This "urban heat island" effect can increase local evaporation and precipitation when combined with the increases in surface water from human outdoor water use[42].

Precipitation, however, cannot infiltrate the predominantly paved and covered urban surface. In Tokyo, 82% of the surface is covered by buildings, concrete, or asphalt, which dramatically decreases infiltration, soil moisture, and groundwater recharge[30]. Consequently, urban precipitation is shunted out of metropolitan areas in larger runoff events and floods. Reductions in groundwater recharge are accompanied by heavy groundwater extraction for urban use, leading to subsidence of some megacities at alarming rates. Parts of Bangkok sank 1.6 m between 1960 and 1988, averaging 5.7 cm/year[28]. This pathology has been documented nicely in Mexico City. An urban heat island of as much as 7.8°C contributes to an intensification of rain showers in the city[43,44]. However, the impermeability of the surface and the large drawdown of the water table has led to parts of the city sinking up to 9 m since 1910[45].

Urban waterways in megacities are polluted enough to pose health hazards to residents, both human and otherwise. Most megacity surface waters have high concentrations of metals, bacteria, suspended solids, ammonia, and phosphates[46,47,48,49,50,51]. Most of these pollutants re-enter waterways from the urban waste stream via leakage of sewerage, leaching of landfills, and basic lack of sewage treatment. In Seoul, one of the world's largest landfills produces 2,600 $m^3$ of leachate per day[52]. Some of the health risks are not due to human pollution, however. In Dhaka, groundwater is naturally rich in arsenic, and most of the urban population is at risk for arsenic poisoning[53].

Poor air quality in megacities has become cliché. The major pollutants produced in urban combustion processes are carbon oxides, nitrogen oxides, sulfuric acids, methane, nonmethane hydrocarbons, soot, and particulates[11,54,55,56,57]. The air chemistry of large industrial cities can be quite complex, and pollution levels are affected by area topography, prevailing wind flows, climate, automotive use, industrial activity, household fuel use, and even animal excrement[58]. Large empirical studies, data analysis, and modeling efforts have been conducted for Los Angeles and, more recently, Mexico City[12]. Because Mexico City is the prototypical modern developing megacity, it serves as a model study system for urban atmospheric research[59,60]. Health risks owing to air pollution are well known[11,54,61,62]. However, the driving force behind air quality policies is more often concern over visibility than health[63].

## Regional Impacts

Regional impacts of the world's largest cities result from the high resource appropriation and waste output rates that are required to satisfy the vast appetites of these megalopolises. Whereas regional economic impacts of major urban areas have been recognized for centuries, the environmental impacts are just beginning to be delineated. Primary among them are the extraction of water from regional watersheds and the polluting of downstream and downwind areas.

Owing to considerations of quantity, quality, and economics, some megacities cannot satisfy their water needs solely from urban waterways and groundwater wells. Wealthier cities such as New York, Los Angeles, Tokyo, Mexico City, and São Paulo rely on piping fresh water in from regional reservoirs and lakes[45,64,65,66], whereas less developed cities simply suffer from grossly inadequate water supplies (see above). In New York, the city has entered a contractual agreement with the regional authorities from which they draw 90% of their water[67]. The security of New York City's water now legally entails the environmental protection of large pieces of regional watersheds. São Paulo is undergoing similar community-based planning to ensure the conservation of watersheds that supply the city's water[66]. These agreements pose interesting trade-offs for regional ecosystems. On one hand, they promise to protect water quality and increase environmental conservation efforts. On the other hand, they have the potential to modify regional hydrology through water extraction and building dams to create reservoirs.

Downstream and downwind impacts are not as potentially beneficial. Wastewater leaving megacities ends up in marine environments (with the notable exception of Mexico City), unnaturally enriching bays and estuaries with nutrients (most importantly nitrogen and phosphorus) that stimulate algal blooms[68,69,70]. The incidence of disease, deformity, and morbidity in marine biota increases due to poisoning from heavy metals[71,72,73,74] and organic compounds[75,76]. Anthropogenic effects also cascade through the trophic web[77,78,79]. A series of studies has documented the physiological, behavioral, and reproductive deterioration of Hudson River tree swallows owing to PCB contamination of their primary food source, aquatic insects[80,81,82]. Heavy marine traffic into megacities and effluent flows induce physical modifications of marine regions such as increased turbidity and sediment loading. Further, megacities are a major source of exotic species introductions, as they are major nodes for global commerce. The 113 documented exotic species in the Hudson River Basin (most likely introduced into New York City) comprise 4 to 60% of well-known groups[78]. The combined effects of anthropogenic activities has altered species composition and community dynamics near several megacities[74,83,84].

Air pollution from megacities has similar effects on downwind aquatic, marine, terrestrial, rural, and urban environments. Nitrogen outputs act as fertilizers for terrestrial environments around Los Angeles[85] and marine habitats worldwide[70]. Oxidants are reducing crop yields in the agricultural areas of densely populated East Asia via direct damage to leaves and reduced sunlight due to increased haze[86,87,88]. The air chemistry of Mexico City has been extensively studied, and remote effects continue to be identified[12]. The Basin of Mexico City generates enough nitrogen oxides, hydrocarbons, and soot to generate plumes of ozone-rich haze that are visible to the naked eye for tens of kilometers and that can even be detected from space[89]. A similar ozone cloud over coastal East Asia is also visible to satellites[90].

## Global Feedbacks

Human activities indisputably affect global biogeochemical cycles, including nitrogen[91,92,93], phosphorus[2,94], sulfur[95,96], carbon[97], and water[94,98]. It is harder to tease out the direct contribution of megacities to global anthropogenic modifications. Nonetheless, very large scale and complex feedbacks now exist between major urban agglomerations and global scale processes. Recent research in Asia provides two examples.

In China, fossil fuel combustion produces oxidizing air pollutants that damage leaf tissue and create a haze so thick it decreases photosynthetically available radiation (i.e., sunlight that plants can use)[86,87,88]. Until recently, China has been virtually self-sufficient for food resources. However, the growing population and reduced crop yield (though for many reasons beyond those mentioned) is increasing the challenge for China to feed its people[99,100]. A bid for self-sufficiency may lead to air quality controls that would otherwise be politically impossible in more democratic states, having a major impact on the environmental quality of the entire region. On the other hand, if China begins to import food, it could become a very influential player in the international food market, affecting agricultural regions around the world.

Another example involves the complex links between motorization and marine ecosystems. Modernization of the highway systems among and within Chinese urban centers is currently a major policy directive and engineering project[29,101]. Construction of motorways and increased motor traffic will suspend large quantities of iron-bearing dust[102]. Acidity attributable to coal burning provides a reducing environment that converts iron to bioavailable forms[103,104]. This could combine with increased nitrogen concentrations owing to fossil fuel combustion[105,106]. Aerosol plumes originating in China are known to cross the Pacific and even circumnavigate the earth[107,108,109]. Because much of the North Pacific is iron limited[110], fertilization of mid-Pacific marine ecosystems is a likely phenomenon[111,112,113]. Increases in marine photosynthetic activity owing to fertilization may then lead to drawdown of atmospheric $CO_2$[114,115]. Zooplankton biomass would increase in response to the boost in primary productivity, thus increasing the sea-to-air flux of dimethyl sulfide, the primary source of cloud condensation nuclei in the open ocean[116]. The cloud condensation nuclei in turn dictate cloud particle sizes and the planetary albedo over the North Pacific, which has the potential to alter global temperatures[117]. Climate change is likely to have significant, yet currently unknown, impacts on Asian winds and hydrological cycles. Thus, it is possible that the development of the Chinese urban infrastructure will induce large-scale climate and biogeochemical effects with serious feedbacks to human activities, both in Asia and globally.

## UNIFYING FRAMEWORKS

Data regarding megacity resource fluxes and environmental factors are abundant but diffuse. Global urban-level data are not systematically collected, nor are they standardized (as they are at the nation level), making analysis, synthesis, and model testing difficult. Successful megacity research will also benefit from conceptual frameworks that help organize and interpret the various geophysical, environmental, economic, and social information. The concepts of urban metabolism and urban succession promise to be quite useful in this regard[12].

### Urban Metabolism

At the most basic level, large metropolitan areas transform fuel and materials into the urban built environment and waste. This closely parallels the process of biotic metabolism, in which organisms absorb or ingest high-energy materials and transform them into biomass, offspring, and waste. The similarities suggest one framework for studying megacities known as urban metabolism[118]. Indeed, the complex behaviors of a large population of humans, all of whom are struggling to obtain energy and do work, create a structurally and functionally whole system that is identifiably a city, just as the specialized cells of an organism form the emergent individual.

An urban metabolism approach calls for the quantification of material and energy budgets, broken down by different ‘metabolic pathways,’ either by element or by industrial sector. For example, the hydrologic path of a city encompasses active (human-driven) inputs such as wells, aqueducts, and pipelines; passive inputs such as rain and groundwater recharge; active outputs such as sewage and storm water flows; and passive outputs such as evaporation and transpiration[12]. The storage and transformation components for water are quite small. Carbon, however, is stored in large quantities as carbonates in infrastructure construction, and it is transformed substantially through the combustion of fuels.

Estimates of the metabolic flows for cities could then be used to identify energy inefficiencies in urban functioning. It is known, for instance, that energy loss in Hong Kong is about 27% within the energy transport infrastructure alone[119]. End-use inefficiencies from idling or inefficient machinery can be even worse; only 49% of the energy that reaches users in Bangalore goes to useful work[120]. It is known that most of the energy consumed by organisms is lost to heat; it is barely possible to make qualitative assessments of urban efficiencies, much less quantitative ones, given the present dearth of data.

Information on the metabolic flows of material and energy through urban areas would permit the quantitative, multidimensional analysis of megacities that is currently lacking in the study of global urbanization. Unfortunately, no complete energy or material budgets exist for any of the world’s largest cities. Of the handful of cities and towns that have been audited, Taipei (population 2.6 million) is the largest[121]. The urban metabolism concept provides an instructive framework for the collection, synthesis, and analysis of static (single point in time) urban data.

## Urban Succession

Similarly, the notion of urban succession would help focus the study of the dynamics of urban development[12]. In wild ecosystems, succession involves the growth and maturation of a biotic community that increases in biomass until it maximally utilizes available energy and nutrients[122]. At this climax stage, biomass is approximately constant, production equals respiration, and the system is at steady state. The ecological evidence suggests that such ecosystems are the very definition of sustainability, a concept that is currently very popular in development, planning, and policy communities.

Contrasting wild and human ecosystem succession highlights ways in which urban systems could begin to approach sustainability. Urban systems undergo a similar process of infrastructure and capital accumulation as long as energy and resources are still available. For example, there is evidence that cities climb the “energy ladder” from a predominance of dirty fuels such as coal to cleaner fuels and more electricity distribution[123,124,125]. Whereas the three primary limitations to wild ecosystem development are light, water, and nutrients, the only real limit to urban growth is currently land area, whose effects are most pronounced for island cities such as Hong Kong and Singapore. (Many major coastal metropolises that are somewhat space-limited are adding land mass by filling estuaries and bays, commonly with trash.) Both urban and wild ecosystems increase in structural and process complexity. However, through time, wild ecosystems generate more cyclic biogeochemical pathways and become better able to retain and recycle nutrients; biogeochemical paths in urban systems, on the other hand, remain fairly straight and exhibit little recycling, large throughput of materials, low total efficiencies, and greater waste production[12]. In most developed cities with centralized water supplies, for example, rainwater is considered a nuisance and is shunted through drain systems out of the city, even though these same cities often suffer water shortages[126].

One interesting difference between wild and human ecosystems is the domination of cities in generating new information for all human societies. Cities can be seen as sources of information and technology that cannot readily be made in rural areas, but which must be imported. This is, perhaps, akin to how photosynthesis introduces nutrients to biotic systems that are otherwise unavailable to many forms of life. Neither light nor ideas seem to be in short supply, but the harvesting and incorporation of both is limited by other factors such as material resource constraints.

Exploring such differences, we believe that urban systems are still far from a climax state. Interestingly, premodern cities were likely in steady state with their immediate surroundings (i.e., their hinterland), which they relied on for renewable resources and waste assimilation and recycling[127]. Megacities, in contrast, obtain resources from around the globe. If modern urban systems are undergoing succession, the climax will occur when global energy sources are maximally utilized, energy flux is at steady state, and infrastructure growth has ceased. This prediction warrants further investigation.

## CONCLUSIONS

With nearly 3 billion people living in urban areas, understanding the biogeochemical processes of cities is imperative for facing the social, ecological, economic, and energy challenges of this century. The functioning, impact, and limits of the world’s largest cities remain largely unknown. Further, it is far from clear how many megacities the earth can support, and for how long. Elsewhere, we have outlined a research agenda for the urban ecosystem that focuses on the role of megacities in the earth system[128]. This research on urban metabolism and succession will be crucial for understanding, predicting, and effectively responding to current and future societal challenges. Megacities will and should continue to draw the scrutiny of environmental and social scientists, biogeochemists, and policy makers.
